# Surface alterations and compound release from aligner attachments in vitro

**DOI:** 10.1093/ejo/cjae026

**Published:** 2024-06-17

**Authors:** Anna Iliadi, Sevasti-Kiriaki Zervou, Despina Koletsi, Marc Schätzle, Anastasia Hiskia, Theodore Eliades, George Eliades

**Affiliations:** Department of Biomaterials, School of Dentistry, National and Kapodistrian University of Athens, Greece; Faculty of Medicine and Health Technology, University of Tampere, Finland; Photo-Catalytic Processes and Environmental Chemistry, Institute of Nanoscience and Nanotechnology, National Centre for Scientific Research ‘Demokritos’, Athens, Greece; Clinic of Orthodontics and Pediatric Dentistry, Center for Dental Medicine, University of Zurich, Switzerland; Meta-Research Innovation Center at Stanford (METRICS), Stanford University, CA, USA; Clinic of Orthodontics and Pediatric Dentistry, Center for Dental Medicine, University of Zurich, Switzerland; Photo-Catalytic Processes and Environmental Chemistry, Institute of Nanoscience and Nanotechnology, National Centre for Scientific Research ‘Demokritos’, Athens, Greece; Clinic of Orthodontics and Pediatric Dentistry, Center for Dental Medicine, University of Zurich, Switzerland; Department of Biomaterials, School of Dentistry, National and Kapodistrian University of Athens, Greece

## Abstract

**Aim:**

The aim of the present study was to assess the alterations in morphology, roughness, and composition of the surfaces of a conventional and a flowable composite attachment engaged with aligners, and to evaluate the release of resin monomers and their derivatives in an aqueous environment.

**Methods:**

Zirconia tooth-arch frames (*n* = 20) and corresponding thermoformed PET-G aligners with bonded attachments comprising two composite materials (universal—C and flowable—F) were fabricated. The morphological features (stereomicroscopy), roughness (optical profilometry), and surface composition (ATR-FTIR) of the attachments were examined before and after immersion in water. To simulate intraoral use, the aligners were removed and re-seated to the frames four times per day for a 7-day immersion period. After testing, the eluents were analyzed by LC-MS/MS targeting the compounds Bis-GMA, UDMA, 2-HEMA, TEGDMA and BPA and by LC-HRMS for suspect screening of the leached dental material compounds and their degradation products.

**Results:**

After testing, abrasion-induced defects were found on attachment surfaces such as scratches, marginal cracks, loss of surface texturing, and fractures. The morphological changes and debonding rate were greater in F. Comparisons (before-after testing) revealed a significantly lower Sc roughness parameter in F. The surface composition of the aligners after testing showed minor changes from the control, with insignificant differences in the degree of C = C conversion, except for few cases with strong evidence of hydrolytic degradation. Targeted analysis results revealed a significant difference in the compounds released between Days 1 and 7 in both materials. Insignificant differences were found when C was compared with F in both timeframes. Several degradation products were detected on Day 7, with a strong reduction in the concentration of the targeted compounds.

**Conclusions:**

The use of aligners affects the surface characteristics and degradation rate of composite attachments in an aqueous environment, releasing monomers, and monomer hydrolysates within 1-week simulated use.

## Introduction

The escalating aesthetic demands exhibited by patients have significantly increased the popularity of orthodontic treatment with clear aligners [1, 2]. To achieve precise crown orientation during aligner treatment, the bonding of resin composite attachments is indicated to enhance rotational control of the teeth. The attachments used are based on conventional or flowable particle-reinforced dimethacrylate resin technology [3]. Each aligner is commonly used for either 1 or 2 weeks and thereafter it is replaced by its sequential successor. With time, the aligner structural instability in combination with residual stress relaxation due to intraoral aging results in decay of the forces applied to teeth [4]. Increased abrasion of the aligner surface in contact with the attachment evidently occurs, which is further accentuated by the removal and re-seating of the appliance, as a common procedure of aligner treatment. In addition, abrasive wear of the composite attachments is anticipated, which is associated with changes in surface morphology [5] and possibly the pattern of applied forces. Considering the popularity of aligner treatment, especially in young patients, and that composite attachments are placed in many aligner cases, related concerns on the safety of the composite compounds released in the oral environment have raised awareness for such treatments. An assessment of the compounds released is considered of priority since the unbonded to bonded surface area ratio of the attachments exposed to the environment is much greater than in most conventional restorative applications at buccal tooth surfaces, while they are subjected to the aligner placement and removal forces of aligners, which create a stressful environment.

The aim of this study was (i) to assess alterations in the morphology, roughness, and composition of the surfaces of a conventional and a flowable composite attachment engaged with the aligners and (ii) to evaluate the release of resin monomers and their degradation products after storage in an aqueous environment. The null hypothesis was (i) that there are no differences in the morphology roughness and composition between the composite attachments before and after 7 days of simulated use, and (ii) there are no differences in the type and amount of the eluents released after 1- and 7-days water storage.

## Materials and methods

### Specimen preparation

Zirconia CAD/CAM upper tooth-arch frames (5Y-TZP outer layer/3Y-TZP inner layer, Upcera Multilayer Dental Zirconia, Shenzen Upcera Dental Technology, Shenzen, Guangdong, China/A1 shade, *n* = 20) and the corresponding thermoformed polyethylene terepthalate glycol (PET-G) aligners (Clear Aligners, Scheu-Dental, Iserlohn, Germany) were manufactured. On each frame, eight resin attachments were bonded on the labial surfaces of central and lateral incisors, canines and first premolars. The zirconia surfaces were grit-blasted (50 μm alumina, 0.23 MPa pressure, 5 mm distance, 90° angle) with an intraoral sandblaster (Microetcher IIA, Danville Materials, S. Ramon, CA, USA) and then treated with a universal primer and an adhesive. Half of the attachment templates were filled with a conventional universal composite restorative material (C), whereas for the rest, a low-shrinkage universal flowable resin composite (F) was employed. The materials used are listed in Table 1. Attachment templates were light-cured for 20 s (each attachment) with an LED curing unit emitting 1180 mW/cm^2^ intensity (Bluphase G2, Ivoclar Vivadent) as measured with a curing radiometer (Bluephase 2, Ivoclar Vivadent). Each frame with the attachments and aligner was stored in 50 ml distilled water in individually sealed glass beakers at 37^o^C under dark conditions. Eight aligners of each group were removed and re-seated to the zirconia frames four times per day for a 7-day immersion period in the beakers using forceps (hereinafter referred to as tested).

**Table 1.** The materials used for composite attachment fabrication.

Product	Type	Composition	Manufacturer
Monobond Plus	Universal primer	MPTMS, 10-MDP, disulfide dimethacrylate, ethanol	Ivoclar Vivadent, Schaan, Liechtenstein
Adhese Universal	Universal adhesive	10-MDP, 2-HEMA, BisGMA, MCAP, D3MA, highly dispersed silica, ethanol, water, photoinitiators (pH = 2.5 − 3)	Ivoclar Vivadent, Schaan, Liechtenstein
Tetric Evo Ceram	Conventional resin composite	Bis-GMA, UDMA, Bis-EMA, inorganic and prepolymer fillers (75-76 w%, 53-55 v%), catalysts, stabilizers, pigments	Ivoclar Vivadent, Schaan, Liechtenstein
Tetric Power Flow	Flowable resin composite	Bis-GMA, UDMA, Bis-EMA, aromatic dimethacrylate, DCP, inorganic and prepolymer fillers (71 w%,) catalysts, stabilizers, pigments	Ivoclar Vivadent, Schaan, Liechtenstein

MPTMS: γ-methacryloxypropyl trimethoxysilane, 10-MDP: 10-methacryloyloxydecyl dihy-drogenphosphate, 2-HEMA: 2-hydroxyethyl methacrylate, BisGMA: Bisphenol-A glycidyl dimethacrylate, MCAP: methacrylated carboxylic acid polymer, D3MA: decandiol dimethacrylate, UDMA: urethane dimethacrylate, Bis-EMA: ethoxylated bisphenol-A glycidyl dimethacrylate, DCP: tricyclodecane dimethanol dimethacrylate.

### Morphological features and roughness

The bonded attachments to the zirconia frames before and after testing were examined under a stereomicroscope (M80, Leica Microsystems, Wetzlar, Germany) at 7.5 × or 25 × magnification under reflected light. Furthermore, the attachments before and after testing were examined by an optical profiler (Wyko NT1100, Veeco, Tuscon, AZ, USA) at 10 × magnification (462.2 × 607.5 μm^2^ analysis area), vertical scanning mode, 2% modulation and tilt correction. The 3D-roughness parameters determined were Sa (arithmetic mean height, the absolute values of the surface height deviations measured from the best fitting plane), Sdr (developed interfacial area ratio, the percentage difference between the true and the projected surface area), Sds (summit density, the number of peaks per unit area of the surface), Ssc (mean summit curvature, the shape and size of the higher areas of a surface) and Sc (core void volume, the volume the surface would support from 10% to 80% of the bearing ratio), and Sv (surface void volume, the volume the surface would support from 80% to 100% of the bearing ratio) [6].

### Composition of exposed attachment surfaces

The molecular composition of tested attachment surfaces engaged with the aligners and their controls was evaluated by attenuated total reflection FTIR spectroscopy (ATR–FTIR). Randomly selected tested specimens (*n* = 10/product) were carefully debonded from the zirconia frames using a straight cutter plier with a torque motion, rinsed with water (10 s), air-dried with an air syringe (20 s), and the central regions facing the aligner were pressed via a sapphire anvil against a single-reflection diamond crystal (2 × 2 mm) of an ATR accessory (ZnSe lenses, 45° incidence angle; Golden-Gate MKII, Specac, Oprington, Kent, UK) attached to an FTIR spectrometer (Spectrum GX, Perkin-Elmer, Buckinghamshire, Bacon, UK). Another series of non-bonded specimes (*n* = 10/product) polymerized in the aligner slots as before and stored for 7 days at 37 °C (dark/dry) were used as control. Spectra were recorded under the following conditions: 4000–650 cm^−1^ wavenumber range, 4 cm^−1^ resolution, 20 scans co-addition and ≈2 μm sampling depth at 1000 cm^−1^. Furthermore, the degree of C = C bond conversion (DC%) of the attachments was measured employing spectra of unset materials as reference. The DC% was calculated based on the two-band technique according to the equation: DC% = 100 × [1 – (*A*p_C=C_ × *A*m_Ar_)/ (*A*m_C=C_ × *A*p_Ar_)] where, *A* is the net absorbance height of the set (p) and unset (m) peaks of the methacrylate C = C bonds stretching vibrations at 1636 cm^−1^ (analytical band; change after photopolymerization) and Ar the aromatic bond stretching vibrations at 1608 cm^−1^ (reference band; not affected by photopolymerization), to compensate photometric errors due to changes in the refractive index of the specimens after setting.

### Determination of compounds released

#### Targeted analysis

Samples of Days 1 and 7 water-eluents were stored at − 20 °C and then analyzed. Blank water solution, eluents from zirconia frames and eluents from zirconia frames with aligners, but without attachments, were used as controls. Targeted analysis of eluents for the determination of Bis-GMA, TEGDMA, UDMA (composites) and 2-HEMA (adhesive) was performed by liquid chromatography-electrospray ionization-tandem mass spectrometry (LC-ESI-MS/MS), using a TSQ Quantum Discovery Max triple-stage quadrupole mass spectrometer coupled to a Finnigan Surveyor LC system, equipped with a Finnigan Surveyor AS autosampler (Thermo Fisher Scientific, Waltham, MA, USA) and a chromatographic column (Atlantis T3, 2.1 mm × 100 mm, 3 μm, Waters, Wexford, Ireland). Analytical standards of the Bis-GMA (≥99%, Sigma-Aldrich, Saint Louis, MO, USA), triethylene glycol dimethacrylate, TEGDMA (99.3%, Sigma-Aldrich, Saint Louis, MO, USA), UDMA (98.9%, Biosynth Carbosynth, Compton, UK), and 2-HEMA (97.6%, A2S, Saint Jean d’Illac, France) monomers (98.9%, Biosynth Carbosynth, Compton, UK) were used to prepare stock solutions (1 mg/ml in methanol) which were stored at −20 °C. Working standard solutions were prepared in the range of 0.25–100 μg/l in water (MilliQ, Merc, Darmstadt, Germany). The chromatographic separation was performed using an isocratic elution (65% acetonitrile–35% water, both containing 0.1% formic acid), employing 0.2 ml/min flow rate, 15 μl injection volume, and 30°C column temperature. The ionization conditions were 4250 V spray voltage, 20 a.u. sheath gas pressure, 5 a.u. auxiliary gas pressure, and 325°C capillary temperature. Detection was carried out in multiple reaction monitoring mode (MRM) using the four most intense and characteristic precursor/product ion transitions, with collision pressure set at 1.5 mTorr. The most intense product ion was chosen to be the quantifier ion, while quantification was performed using external standard calibration. Monomer identification was based on retention time, four characteristic precursor/product ion transitions, and three calculated ratios of precursor to product ion transitions. The analytical performance of the method was evaluated by assessing the linearity and range of measurement, specificity, precision, and limit of detection (LOD). Standard solutions were prepared at seven different concentrations (0.25, 0.5, 1, 10, 25, 50, and 100 μg/l) of monomers and analyzed in triplicates to evaluate linearity and measurement range. To assess the specificity, blank samples were analyzed to assess any interferences close to the retention time of the monomers. Method precision was assessed by repeated measurements (*n* = 7) of standard monomer solutions at the concentration levels of 1 and 100 μg/l, whereas the LOD of the method was validated by carrying out analysis of spiked samples (*n* = 5) at the concentration of 0.25 μg/l.

For BPA, considered as impurity or degradation product of BPA adducts, analytical standard (≥99%, Sigma–Aldrich, St. Louis, MO, USA) and internal standard (IS) of the isotopic labeled BPA-d16 (98.1%, Dr Ehrenstorfer GmbH, Augsburg, Germany) were used to prepare stock solutions of each compound (1 mg/ml in methanol) which were stored at −20°C. All the samples were spiked with BPA-d16 (IS) at final concentration of 50 μg/l, prior to analysis. Calibration solutions of BPA, at an IS concentration range from 0.25 to 75 μg/l with 50 μg/l in water, were prepared and analyzed. The analysis was performed by liquid chromatography–atmospheric pressure chemical ionization–tandem mass spectrometry (LC–APCI–MS/MS). Chromatographic separation was carried out with an isocratic elution employing 50% acetonitrile–50% water mobile phase, 15 μl injection volume, and 30 °C column temperature. Ionization conditions were set as follows: 4.0 V discharge current, 400 °C vaporizer temperature, 25 a.u. sheath gas pressure, 15 a.u auxiliary gas pressure, and 350 °C capillary temperature. Detection of BPA and IS was performed in MRM mode using the four and two most intense and characteristic precursor/product ion transitions, respectively, with collision pressure set at 1.5 mTorr. The performance of the analytical method was evaluated, as previously, by assessing the linearity and range of measurement, specificity, precision, and LOD. Standard solutions at eight different concentrations (0.25, 0.5, 1, 5, 10, 25, 50, and 75 μg/l) of BPA with 50 μg/l IS were analyzed in triplicates, to evaluate linearity and range of measurement. For the assessment of specificity, blank samples were analyzed for the presence of peaks interfering with the retention time of BPA. Method precision was assessed by repeated measurements (*n *= 7) of BPA standard solutions at the concentration levels of 0.5 and 50 μg/l and for the LOD analysis of spiked samples (*n *= 3) at the concentration of 0.25 μg/l was performed. For targeted analysis data processing was performed using Xcalibur software 2.1 SP 1160 (Thermo Fisher Scientific).

#### Untargeted analysis

Suspect screening and untargeted analysis of the eluents was performed with an Orbitrap Q Exactive Plus high-resolution mass spectrometer (HRMS), coupled to an Ultimate 3000 series LC pump and autosampler (Thermo Fischer Scientific). Chromatographic separation of the compounds was carried out with the same column. The mobile phase was (A) water and (B) 90% acetonitrile–10% water both containing 5 mM ammonium formate and 0.02% formic acid. The gradient program started at 5% B (held for 3 min), increasing to 65% B in 3 min (held for 4 min) and to 90% B in 10 min (held for 10 min). The flow rate was set at 0.2 ml/min with 10 μl injection volume and the column temperature was set at 30 °C. Compounds were ionized using an ESI source operated in positive mode. Data acquisition was performed in data dependent mode (DDA). Full MS spectra were obtained in Orbitrap with a resolution of 70 000 and scan range *m*/*z* 70–1050. The four most intense ions of the full scan were fragmented with a stepped collision energy of 10, 30, and 50. The isolation window width was 1 Da and the resolution was set at 17 500 for the acquisition of the fragmentation spectra. Data processing was performed using Compound Discoverer 3.2TM software (Thermo Fisher Scientific, Waltham, MA). A data processing workflow was developed, in which an in-house mass list of 85 compounds correlated to dental resins and the mass list of extractables and leachables HRMS compounds were incorporated. By the applied workflow, features were extracted from the experimental spectra with 3 ppm mass tolerance, aligned according to the retention time, and the full scan (MS1) spectra were grouped with the corresponding fragmentation (MS2) spectra. Annotation of the compounds from the mass list was performed based on the exact mass with 3 ppm mass tolerance, isotopic pattern match of elemental composition, manual check of MS2 spectra, and comparison to the available fragmentation data from the literature [7, 8]. Additionally, a search on mzCloud [9], for untargeted screening was performed. The search on mzCould was part of Compound Discoverer data processing workflow. This search enabled compound annotation based on the similarity of fragmentation spectra. All the compounds were tentatively identified without native standards by using MS data at a confidence level 2 for metabolomics analysis [10].

### Statistical analysis

Data distribution was checked for all tested roughness parameters statistically through Shapiro-Wilk tests and visually through *q*–*q* plots. For roughness measurements Wilcoxon signed rank tests were used to examine differences between control and tested groups (before-after) within each composite material. Mann–Whitney tests were used to identify differences between the two materials (C and F) for both control and tested groups, as appropriate. For DC% measurements, Student’s *t*-tests were used for comparisons before and after testing per material and between materials for the two conditions.

For the compounds released, samples were determined based on their proportion within three categories according to the level of detection and quantification of the monomers as follows: (i) below the level of detection (< LOD), (ii) below the level of quantification (< LOQ), and (iii) above the level of quantification (> LOQ). The distribution of data for quantifiable monomers (> LOQ) was checked statistically through Shapiro–Wilk tests and visually through *q*–*q* plots. Median and interquartile ranges were assessed. Fisher’s exact tests were performed as applicable to identify statistically significant differences between Days 1 and 7 of examination of the samples for each monomer across type of composite (C or F). Likewise, Fisher’s exact tests were also applied to record the effect of composite on each monomer categorization in both examination timeframes (Day 1, Day 7). Ordinal logistic regression was built to capture statistically significant differences between monomers, according to the 3-level concentration categorization, per composite type and timeframe. The level of statistical significance was determined at *P* < .05 (two- sided alpha on 5%). All analyses were performed using Stata version 15.1 (Stata Corp. College Station, TX, USA).

## Results

### Morphological features and roughness

**Figure 1.**
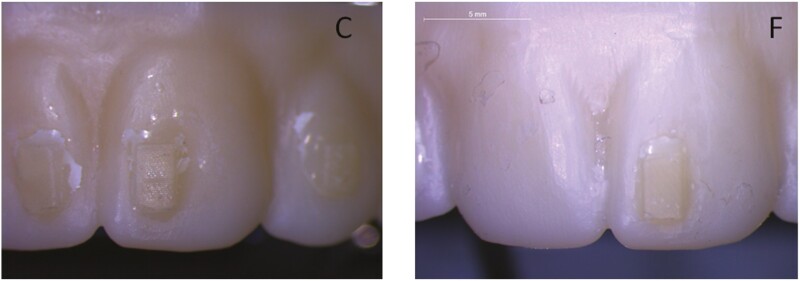
Stereomicroscopic images of zirconia frames with composite attachments after testing. Note attachment debonding in F (7.5 × magnification, bar: 5 mm).

**Figure 2.**
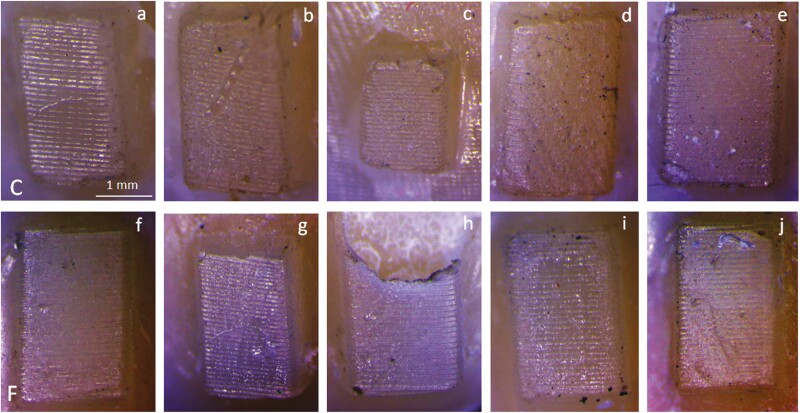
Stereomicroscopic images of zirconia frames with composite attachments (C: upper row; F: lower row) after testing. Note surface scratches (a, b for C, all for F), marginal defects (c, e, f, g, j), pronounced surface abrasion with loss of the characteristic surface texture (d, f, j), and bulk fractures (h). Top part of images: cervical region, bottom part: incisal region (25 × magnification, bar: 1 mm).

Representative stereomicroscopic images of zirconia frames with composite attachments after testing are illustrated in Fig. 1. The attachment debonding rate after testing was 14.1% in C and 31.3% in F. Morphological features of C and F attachments before and after testing are presented in Fig. 2. The control groups demonstrated a textured surface morphology, with no marginal defects. The post-testing defects included scratches on the attachment surfaces, marginal defects mainly at the cervical regions with fracture or rounding of the attachment edges and angles, and in some cases loss of the characteristic surface texturing. A higher frequency of texturing loss and bulk attachment fractures was found in F attachments.

**Figure 3.**
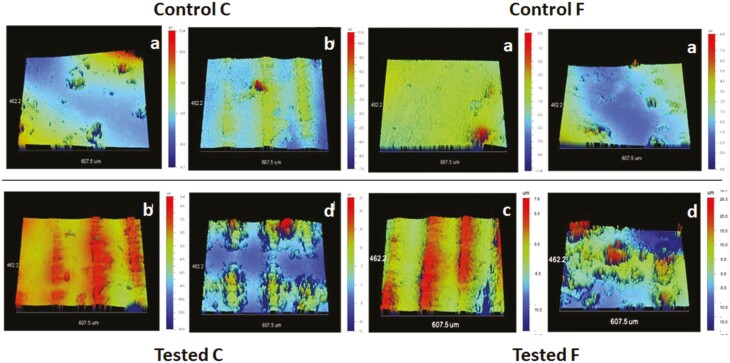
3D-profilometric images of Groups C and F (control vs tested). The surfaces demonstrate mild porous defects (a), appearance of the texturing of the intaglio aligner surface (b), cracks (c) and severe porosity and abrasion associated with the texturing protrusions (d) (10 × magnification, 462.2 × 607.5 μm^2^ analysis area).

3D-profilometric images of the attachment surfaces are presented in Fig. 3. The abrasion-induced defects included scratches, marginal defects with fracture or rounding at the edges, loss of surface texturing and attachment fractures. The quantitative findings of the roughness parameters are summarized in Table 2. There were no statistically significant differences between control and tested groups per material, except for Sc solely in F, where the tested specimens demonstrated significantly higher values (*P* = .02, before-after: all Wilcoxon signed-rank tests). Comparisons between the control materials of the two composite groups (all Mann–Whitney tests) demonstrated scarce evidence for a statistically significant difference only in Sds in favor of F (*P* = .05). Finally, comparisons between the two tested groups (all Mann–Whitney tests) revealed weak evidence for a significantly lower Sds value in C (*P* = .046).

**Table 2.** Results of roughness parameter measurements before (control) and after (tested) the experimental period across composite types (median and interquartile range in parentheses, C: conventional, F: flowable).

GROUP	Sa (μm)	Sdr (%)	Sds (1/mm^2^)	Ssc (1/mm)	Sc (mm^3^/mm^2^) × 10^3^
Tested C	1.68 (1.28–3.06)	3.92 (2.64-7.10)	1707.67 (1599.98-1850.00) b	285.99 (276.48-446.07)	2.89 (2.19-5.52)
Control C	1.60 (0.71-2.51)	3.14 (0.89-5.50)	1599.47 (1348.92-1937.16) B	248.62 (158.69-306.71)	2.41 (1.22-3.76)
Tested F	1.54 (1.04-2.61)	4.42 (3.49-7.20)	1906.82 (1763.00-2311.44) b	367.45 (311.25-414.08)	2.50 (1.81-4.50) A
Control F	1.14 (0.82-1.63)	4.61 (2.69-5.64)	2142.03 (1986.82-2295.61) B	347.82 (285.77-393.25)	1.56 (1.30-2.06) A

Index A: Statistically significant differences for comparisons between control and tested (before-after) within each material group (control-tested, Wilcoxon signed rank tests for all comparisons, *P* = .02).

Index b: Statistically significant differences for comparisons between materials after testing (C–F, Mann–Whitney tests for all comparisons, *P* = .04).

Index B: Statistically significant differences for comparisons between control materials before testing (C–F, Mann–Whitney tests for all comparisons, *P* = .046).

### Composition of exposed attachment surfaces

**Figure 4.**
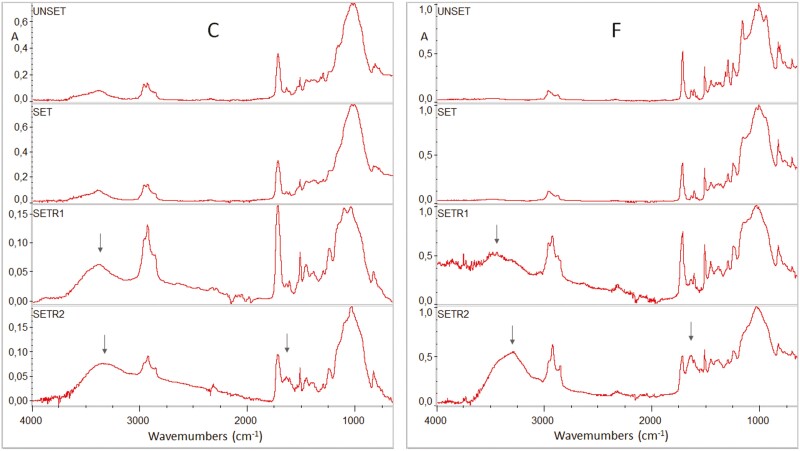
ATR-FTIR spectra of unset and set states of C and F attachments. SETR1 and 2 correspond to set specimens after testing. Arrows show the strong peaks observed in some specimens possibly assigned to carboxyl and water interferences (4000–650 cm^−1^ wavenumber range, absorbance scale).

**Figure 5.**
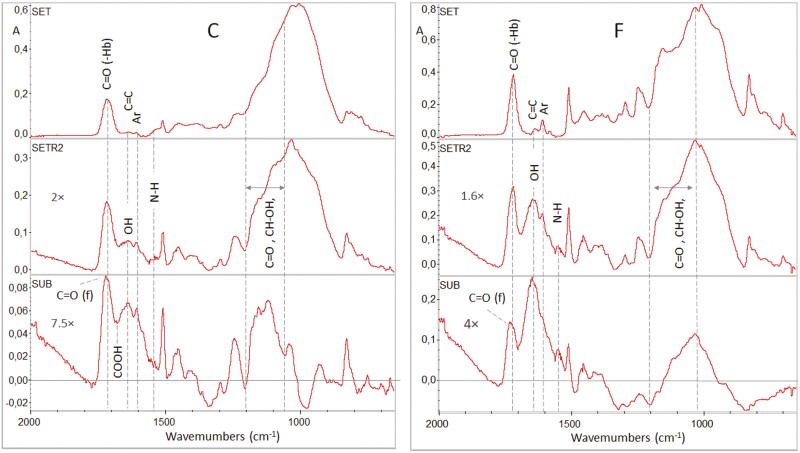
ATR-FTIR spectra of light-cured C and F attachments before (set) and after testing (SETR2) along with the corresponding subtracted spectra (SUB, tested-control). Annotated peaks show the major changes after testing (2000–650 cm^−1^ wavenumber range, absorbance scale with magnification factor).

Full range ATR-FTIR spectra of unset and set C and F specimens along with specimens after testing are presented in Fig. 4. The spectra demonstrate characteristic peak assignments as follows (cm^−1^): O–H (3442, 1140–1110), N–H (3371), aromatic C..C (3010, 1608, 1595,1510, 830, 801), CH_3_/CH_2_/CH (2920–2880, 1465–1430, 1370–1360, 720–700), C = O (1715, 1320, 1290), C = C (1634, 1500, 895), CON–H (1540), C–O–C (1260, 1105–1000), and Si–O (1150–1000) [11]. These are the common peaks identified in composites with conventional bisphenol-A adducts (i.e. BisGMA, BisEMA), triethyleneglycol dimethacrylate (i.e. TEGDMA) and urethane dimethacrylate co-monomers (i.e. UDMA, DUDMA). After testing, some specimens showed strong and broad base peaks at the 3450–2800 cm^−1^ band range and at 1642 cm^−1^, assigned to carboxylic acid dimers and water [12]. Spectra of specimens with such interferences before and after testing along with their subtraction spectra (after–before) at the fingerprint region (1700–650 cm^−1^) are illustrated in Fig. 5. For both attachment materials a high wavenumber shifting is observed in C = O peaks (from 1718 to 1733 cm^−1^) indicating reduced extent of H-bonding as it is clearly documented in subtraction spectra. Strong O–H interferences appear at 1642 cm^−1^, masking off the C = C peak (1638 cm^−1^), with increased intensities of aromatic (1605 cm^−1^) and amine (1540 cm^−1^) peaks. At the major complex peak region (1200–800 cm^−1^), where absorptions of C = O, C–O, C–C, C = C, CH–OH, Si–O (filler particles) lie, the tested specimens demonstrated an increase in the high wavenumber fraction (mainly C = O, CH–OH, and C–O groups). All these changes were more pronounced in F. Specimens with water interferences overlapping the analytical C = C band were excluded from the degree of C = C conversion measurements (DC%).

Table 3 summarizes the results of DC% for the groups tested. Comparisons between control and tested groups per material, between the control groups and between tested groups showed statistically insignificant differences (*P* > .05, all Student’s *t*-tests).

**Table 3.** Descriptive statistics on the degree of conversion (%DC) measurements before (control) and after (tested) the experimental period across composite types (means and standard deviations in parentheses). There are no statistically significant differences (*P* > .05).

Group	DC (%)
Tested C	69.7 (8.8)
Control C	62.6 (1.9)
Tested F	66.2 (4.8)
Control F	61.5 (1.1)

### Determination of compounds released

**Figure 6.**
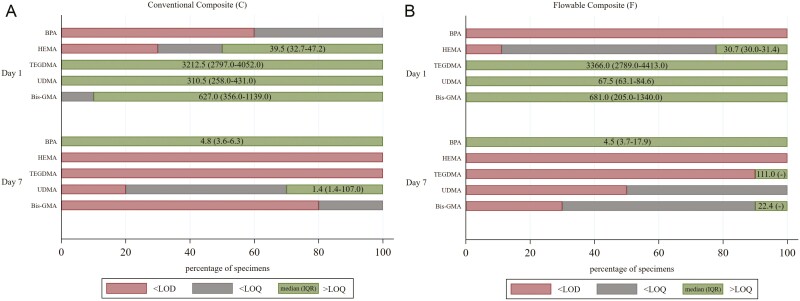
Graph bars of the targeted analysis for the compounds probed in eluents (median and interquartile range in μg/l) expressed in percentage of specimens for the two timeframes (Days 1 and 7). LOD: limit of detection, LOQ: limit of quantification. LOD (μg/l): BPA (0.25), HEMA (10), TEGDMA (0.5), UDMA (0.25), Bis-GMA (5). LOQ (μg/l): BPA (1), HEMA (30), TEGDMA (1.5), and UDMA (1), Bis-GMA (15).

**Figure 7.**
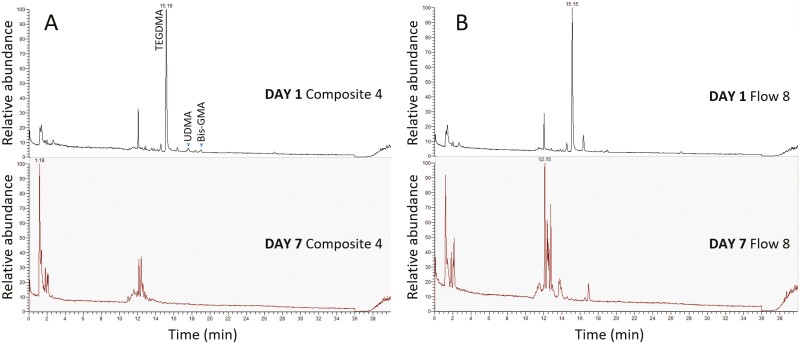
Full scan chromatograms of C (sample #5, A) and F (sample #8, B) composite eluents. The peaks of TEGDMA, UDMA and Bis-GMA are missing from Day 7 eluents and new unknown peaks have been amplified.

Representative full scans chromatographs of eluents are presented in Fig. 6. The findings of targeted analysis by LC-MS/MS are presented in Fig. 7. The blank water solution, the eluents from zirconia frames and the eluents from zirconia frames with aligners without attachments, which served as controls, did not reveal any presence of interfering chemical compounds above the detection level. TEGDMA and UDMA were fully quantifiable on Day 1, with median concentrations between 3212.5 μg/l for C and 3366.0 μg/l for F regarding TEGDMA, and 310.5 μg/l for C, 67.5 μg/l for F regarding UDMA. Bis-GMA was above level of quantification (> LOQ) in F with a median value of 681.0 μg/l, and in 90% of C samples, with a median value of 627 μg/L. 2-HEMA was >LOQ in 50.0% of C and 22.2% of F specimens (30.7–39.5 μg/l median values). On Day 7, the only quantifiable monomers (>LOQ) were UDMA (30.0% of C specimens), and TEGDMA, Bis-GMA (10.0% of F specimens). BPA, was not detected or detected below the LOQ for both composites (C and F) on Day 1, while on Day 7, BPA was quantifiable in all cases with a median concentration of 4.8 μg/l for C and 4.5 μg/l for F. A statistically significant difference was confirmed for each examined compound between Days 1 and 7 (Fischer’s exact test, *P* < .004, all comparisons) in both materials tested. No statistically significant difference was detected for any of the compounds when C was compared with F in both timeframes (Fischer’s exact test, *P* > 0.05 all comparisons). Between compounds, comparisons in terms of distribution < LOD, < LOQ, or > LOQ, within the same timeframe and composite, did not reveal significant differences (ordinal logistic regression, *P* > .05).

**Figure 8.**
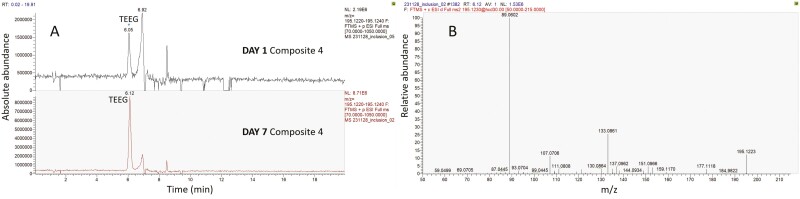
Example of untargeted analysis for TEEG (transformation product of TEGDMA). A: Extracted ion chromatograms of *m*/*z* 195.123 that corresponds to [M + H]^+^ of TEEG (Days 1 and 7 eluent of C, sample #4). B: Fragmentation spectrum of TEEG with precursor ion *m*/*z* 195.123. Note the increased intensity of TEEG peak in the chromatogram on Day 7.

**Figure 9.**
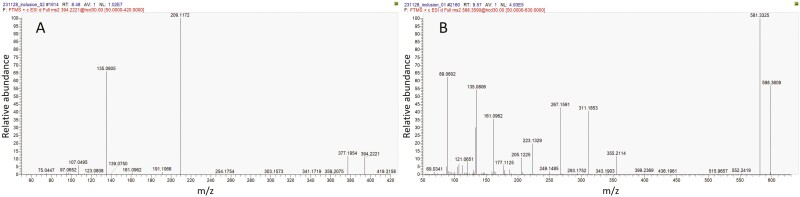
Fragmentation spectra of BisHPPP (A; precursor ion [M + NH_4_]^+^*m*/*z* 394.2221) and of BisE-08 (B; precursor ion [M + NH_4_]^+^*m*/*z* 598.3590), both degradation products of Bis-GMA, detected in the eluents on Day 7.

Representative results of untargeted analysis are illustrated in Figs. 8 and 9. Transformation products of TEGDMA, such as tetraethylene glycol (TEEG, four times higher abundance in C on Day 7 than Day 1), triethyleneglycol monomethacrylate (TEGMMA), triethanolamine and triethylene glycol (TEG) were found on Day 1 or on Days 1 and 7, without a significant difference (Fig. 8). The degradation products of Bis-GMA, ethoxylated bisphenol A glycol-7 and -8 (BisE-07, BisE-08) were detected in F on Day 7, whereas the degradation product Bisphenol A, bis(2,3-dihydroxypropyl) ether (Bis-HPPP) was detected in C and F on Day 7 (Fig. 9). Moreover, 4-(dimethylamino) benzoic acid (DMAB), the degradation product of the photoinitiator ethyl 4-dimethyl-aminobenzoate (EDMAB) contained in C and F was detected in C eluents on Day 7. The monomethacrylate adduct of UDMA (UDMA-D1) was also found in the 1-day eluents of both materials.

## Discussion

The results of the present experimental study showed that there were differences in the surface morphology of the two types of composite attachments (conventional and flowable) after repeated aligner placement and removal in comparison with the control (before testing). However, no statistically significant differences in the roughness parameters, except for Sc in F, were found before and after testing. The composition of the aligner surfaces, in most cases, showed minimal changes associated with water interferences and insignificant DC% differences from the controls. Nevertheless, few specimens showed evidence of severe hydrolytic degradation in both composites after a 7-day testing. Therefore, the first null hypothesis should be rejected for morphology and accepted for roughness, composition, and DC%, apart from Sc in F and the limited number of specimens with hydrolytic degradation. The water-eluents of the specimens demonstrated great differences in the targeted compounds between 1- and 7-day testing period in both materials, with many untargeted compounds (mostly hydrolysates of targeted monomers) in 7-day testing. Consequently, the second null hypothesis should be rejected.

The materials selected in the study were a conventional (C) and a flowable (F) universal composite restoratives, commonly used for aligner attachments. The former demonstrates higher viscosity and better mechanical properties due to the higher filler content, whereas the latter is a thixotropic flowable. Although in previous studies highly loaded materials were used for aligner attachments [5, 13], currently, several manufacturers have introduced flowable materials facilitating porous-free application in the attachment inserts of the aligners and intimate contact with tooth surfaces. The substrates simulating tooth bonding surfaces were zirconia (5Y-TZP) frameworks, which offer water insolubility, absence of interfering compounds in the eluent measurements by LC-MS/MS, dimensional stability in water, very low rate of low-temperature degradation and bonding capacity of the composite attachments. The use of low-pressure alumina blasting and a 10-MDP containing primer has been widely accepted as an effective method for strong and durable resin bonding to zirconia [14, 15]. This model provides a standard arch design for comparatively testing similar attachments bonded at the same locations with different composites. The study was limited to one week period, the effective life of aligners, to evaluate the early changes induced in the freshly bonded attachments. Initially attachments are not matured, as post-curing reactions proceed, albeit they are directly exposed to the oral environment and aligner forces. Water was selected as an immersion medium to avoid interferences in the LC-MS analysis with commonly used electrolyte solutions, such as artificial saliva, and to reveal the maximum releasing capacity at a physiologic pH. It has been shown that water demonstrates similar monomer release rates with artificial saliva solutions, but less than the 75% ethanol-water solution originally proposed as a clinically relevant food simulant [16]. The latter induces the highest extent of resin plasticization, increased and prolonged monomer release, with evidence of monomer degradation [16]. It has long been documented that one-week composite water immersion is considered as an absorption equilibration period, where remaining monomer and early oxidative compounds of pendant C = C bonds are released [17] and that the rate of these phenomena is usually reduced over time [18]. In the current study, a decay of the stress-induced attachment corrosion is anticipated, since the attachments may lose conformity with the aligners, reducing interfacial friction [13].

The stereomicroscopic images of the control groups showed evidence of surface porosity and loss of the characteristic texturing of the intaglio aligner surface (a result of thermoforming the aligner material against the 3D-printed moulds) in some specimens, which may indicate inadequate wettability of the aligner by the composite. Such defects were limited in F. In the tested groups the attachment surfaces exhibited cracks, loss of surface texturing and in some cases bulk fractures, more frequently observed in the mechanically weaker F. Although comparison of attachment reproduction by conventional and flowable composite has shown insignificant differences in shape and volume parameters with a ± 0.1 mm tolerance [19], these measurements cannot provide information on the overall surface qualities, especially after aging.

An increased debonding rate was found in F, with more than half failures occurring during the first removal of the aligner, leaving the attachment locked inside the aligner frame. The higher wettability and mechanical retention of F with the textured internal aligner surface and the increased shrinkage in comparison with C [20–22] may have contributed to this debonding mode. Although the clinical relevance of this finding is unknown, it may suggest a potential limitation of the currently available flowable composite materials as aligner attachments. Nevertheless, high-viscosity composites, may demonstrate application and adaptation problems in small aligner inserts designated for attachments. The issue of adaptation precision of aligners to flowable and bulk-fill resin composite rectangular attachments, without in-service aging, has been evaluated by scanning electron microscopy (SEM) on buccolingually sectioned resin casts [23]. The results showed that the best fit was obtained with the highly filled bulk-fill resin composite. Nevertheless, assessment under high vacuum and high accelerating voltage of non-embedded soft and hard polymer interfaces may create artifacts associated with differential thermal effects, which may bias the results.

A range of amplitude (Sa, Sq, Sz), hybrid (Sdr, Sds, Ssc), and functional (Sc, Sv) roughness parameters were selected to better characterize exposed attachment surfaces. The profilometric images obtained, at more than twice the stereomicroscopic magnification, represented the most affected zone. The data used were unfiltered. From the parameters tested, Sdr and Ssc are considered of major importance to evaluate how surfaces interact when the one is moved against the other, how friction is implicated and how they abrade due to the contact [24]. These two parameters focus on the actual contact area due to the presence of surface summits than the entire area, and can be used to predict the mode of surface deformation under load, the friction and wear characteristics of a surface [24]. However, the high variances in topography resulted in statistically insignificant differences between control and tested groups per material, except for Sc in F, which increased after 7-day testing. This indicates that the core volume of the attachment surfaces in F was increased apparently due to abrasive wear. Comparisons within control or tested groups evidenced a significantly higher Sds value for F, albeit weak in statistical terms. This corroborates the improved rheological properties of F which may better penetrate the texturing details of the aligner surfaces facing the teeth.

The analysis of the attachment surface composition was focused on the organic part of composites since the organic compounds are mainly associated with possible side effects. The ATR–FTIR spectroscopic analysis probed the chemical changes within the superficial 2 μm zone of the materials, which is in the range of the Sa values of all the groups tested. In most cases, the spectra of specimens obtained after testing resembled those of the controls, indicating limited evidence of hydrolytic degradation. However, in some cases, chemical changes were observed associated with water absorption, reduction in the binding state of ester H-bonded fraction (a condition vulnerable to hydrolysis), carboxylic acid formation and presence of more intense peaks of aromatic, amine, alcohol, ester, and ether groups. These findings support an increased hydrolytic susceptibility, which may involve two mechanisms; first stress-induced hydrolytic corrosion of the composite surfaces, and second irreversible absorption of low molecular weight hydrolysates from the storage solution, since the species probed at the composite surfaces were not affected by water rinsing and air drying. Under the current experimental conditions, the first mechanism seems the most pertinent, possibly associated with the attachments located at high-stress areas in the dental arch. The degree of C = C conversion is a fundamental property affecting the mechanical, chemical, and biological properties of methacrylate-based dental polymers [25]. The DC% values recorded did not show statistically significant differences between the control and tested groups per material or between materials per condition. The conversion ranged between 61.5% and 69.7%, which is above the limits for composite restorative materials offering an acceptable abrasive wear depth (55%) [26].

The eluent analysis revealed detectable amounts of all the targeted monomers on Day 1, with TEGDMA, Bis-GMA and UDMA being the predominant species. The presence of 2-HEMA indicates minor elution from the universal adhesive. This early release should be mostly assigned to residual monomers, that is reduced over time [18, 27]. On Day 7, the only quantifiable monomers were UDMA for one third of C specimens and Bis-GMA, TEGDMA for even a less fraction of F specimens. TEGDMA demonstrated the highest release rate on Days 1 and 7, a common finding with similar studies, attributed to the small size and hydrophilic nature of the monomer, which creates weaker networks than UDMA, and absorbs more water [28]. Bis-GMA showed higher release than UDMA. Although Bis-GMA is more hydrophobic with higher viscosity than UDMA, the latter demonstrates better conversion and more crosslinking capacity due to reduced steric hindrance [29]. The lower release of UDMA from F may be explained by the presence of two more monomers in F, the aromatic dimethacrylate (possibly propoxylated Bisphenol dimethacrylate) and DCP, according to the manufacturer, which reduce the specific monomer content in the composite. Detection of UDMA-D1 on Day 1 material eluents implies that this may be considered an unstable degradation product which is further transformed, after 7-day storage.

On Day 1 measurements, BPA was detectable only in 40% of C specimen eluents below the limit of quantification. However, on Day 7 quantifiable BPA amounts were traced in both material eluents (4.5–4.8 μg/l), without statistically significant differences. This may imply that either BPA impurities originating from bisphenol adducts increased in concentration after the 7-day storage period, or adduct derivatization occurred during the storage period, creating monomer hydrolysates. BPA could be a possible degradation product of Bis-GMA, formatted in a similar pathway such as in the case of Bis-PMA [30].

The untargeted analysis confirmed the hypothesis of monomer hydrolysis, by annotation of several derivatives of TEGDMA and Bis-GMA, such as TEGMMA, TEEG, TEG, triethanolamine, Bis-E-07, -08 and Bis-HPPP, known as biodegradation products [31–33]. Apparently, in the present study, the eluents hydrolyzed during storage possibly via nucleophilic attack of water to the ester bonds at physiological pH. The final product of hydrolysis of these monomers is formation of carboxylic acids, which may further accelerate the degradation process [32]. The high rate of degradation as documented by the absence of certain monomer eluents on Day 7, may imply that the testing procedure (aligner removal and reseating) strongly contributes to monomer derivatization, considering that in other studies, main monomers were detected even up to 28 days storage in a more aggressive environment (75% ethanol–water) [27].

The *in vitro* nature of the study imposes several limitations, and the results should be carefully interpreted. Although intraorally enzymatic attack may enhance biodegradation reactions, saliva proteins may bind with eluents reducing the *in vivo* availability [16], Moreover, pellicle formation and the lubricating effect of saliva polysaccharides may lower the degradation rate via adsorption and reduce the aligner-composite friction reduction. Nevertheless, intraoral sampling represents a momentary concentration of the eluent, contrary to the *in vitro* model that may provide cumulative results, critical for material development and optimization. In the present study the targeted analysis was focused only on the attachment material and not the aligner, since thermoformed polymers (PET, PET-G, poly ester-urethanes, etc.), are considered more stable than the resinous 3D-printed aligners, which may release additional monomers or degradation products [34].

The biological effects of dental resin monomers on cell viability (death and apoptosis), oxidative stress, DNA damage and regulation, immunomodulation, genotoxicity, and estrogenicity have been the subject of many studies in the relevant literature [35–37]. Recently, important work has highlighted the need for full characterization of the degradation products of resin monomers and their complex biological activity locally and systematically [7, 8, 30]. Considering the caveats associated with the release of resin compounds intraorally [38], the present *in vitro* study highlights for the first time the role of aligners in the high rate of monomer derivatization from composite attachments, that merits further investigation.

## Conclusions

The use of aligners affects the surface characteristics and degradation rate of resin composite attachments in an aqueous environment, releasing resin monomers and, most importantly, monomer hydrolysates within one-week simulated use.

## Data Availability

I have read the journal’s requirements for reporting the data underlying my submission (data policy in EJO Author instructions) and have included a Data Availability Statement within the manuscript. Data are available upon request to the corresponding author.
